# Wireless Lighting System: A New Tool for Assessing Cognitive Functions in the Elderly

**DOI:** 10.3390/bs13110943

**Published:** 2023-11-17

**Authors:** Néva Béraud-Peigné, Alexandra Perrot, Pauline Maillot

**Affiliations:** 1 Complexité, Innovation, Activités Motrices et Sportives, University of Paris Saclay, 91440 Bures-sur-Yvette, France; alexandra.perrot@universite-paris-saclay.fr; 2 Complexité, Innovation, Activités Motrices et Sportives, University of Orleans, 45062 Orleans, France; 3 Institut des Sciences du Sport-Santé de Paris, University Paris-Cité, 75015 Paris, France; pauline.maillot@u-paris.fr

**Keywords:** executive functions, older adults, motor capacity, dual task, validity, test

## Abstract

Studies on the effects of aging on cognition have been using the same cognitive tests for decades. A Wireless Lighting System (WLS) could be used to assess cognitive functions in a physically active situation, making the assessment of cognition less isolated and more ecological. This pilot study aimed to evaluate the potential of a WLS for assessing older adults’ cognitive functions. It was set up with 15 young (M = 23.47 years old) and 18 older adults (M = 71.44 years old). Their performances were recorded on three WLS tests, designed with the *Witty SEM* system to assess four main cognitive functions (i.e., inhibition, flexibility, visuospatial short-term and working memory), as well as on three traditional (TRAD) tests (Spatial Span Test, Stroop Test, Trail Making Test). The results show a significant difference between the YOUNG and OLD groups on all WLS test measures (except for WLS flexibility), as well as on all TRAD measures. Additionally, for the OLD group, there were significant correlations between WLS and TRAD test results (*r* = −0.49 for two measures of inhibition to *r* = −0.80 for two other measures of inhibition), except for visuospatial short-term memory. However, there was no significant correlation for the YOUNG group (ρ = −0.27 for inhibition to *r* = 0.45 for visuospatial short-term memory). These WLS tests were valid for assessing the cognitive functions (i.e., flexibility, visuospatial short-term and working memory, inhibition) of older adults and were sensitive to aging.

## 1. Introduction

The increasing population of older people implies new challenges for society, such as keeping older adults healthy and active for longer. Preventing and/or reducing the risk of physical and cognitive impairment is one of the top public health priorities [1]. Researchers develop new modes of intervention (e.g., cognitive–motor training, multi-domain training), notably with new technologies (e.g., exergames [2], connected bikes [3], video games [4]), to provide optimal cognitive and physical benefits in older adults and also create positive experiences and pleasure. The question of how to evaluate these preventive programs is crucial for knowing their precise effects and being able to perfect them. Additionally, it is essential to develop tests closer to life’s daily demands, so as to identify the level of transfer effect.

Many studies on the effects of aging on cognition have been using the same cognitive pencil and paper tests for decades. For example, the *Trail Making Test* [5], the *Stroop Test* [6], or the *Spatial Span Test* [7] are the gold standard for assessing cognitive functions. Although these tests are validated, reliable, sensitive, and effective in highlighting the effects of programs on the elderly, they have some limitations. How the researcher explains, administers, and encourages may vary depending on the subject. Time measurements are also often used, but triggered and recorded by the experimenter with a simple stopwatch. This can create imprecise measurements. The questions are not randomly generated with each administration, so opportunities for retesting are reduced. In conclusion, traditional tests may not be sensitive and accurate enough. This inadequacy may be one of the reasons why, currently, no systematic differences have been found between the various types of physical or cognitive intervention, with studies using traditional tests with an interventional design (pre-tests—training—post-tests) (for review, see [8]). Finally, researchers may regret the lack of ecological validity of these tests as they do not sufficiently reflect performances in daily life. The level of cognitive functions is closely linked to motor skills in everyday life, yet traditional tests isolate cognitive functions. Adding a motor dimension to these tests would be interesting, especially with the emergence of multi-domain training that solicits cognitive and motor functions. Therefore, developing new modes of evaluation of cognitive functions appears necessary, with sensitive and ecological measurements. Developing new technologies [9] could be the solution, as they would be more efficient at assessing an individual’s abilities than their paper-and-pencil equivalents [9]. Response latencies with millisecond accuracy increase test sensitivity, making computerized tests valuable. The administration and scoring are also automated, enabling researchers to simultaneously collect data, record, and report on many aspects of the performance. It is possible to automatically store and compare a person’s performance between testing sessions. Consequently, the interpretation of results is more straightforward, accurate, and objective [10]. In addition to these points, administrators’ testing biases are limited thanks to the standardization of test administration [11]. Lastly, tests can adapt to the examinee’s level of performance to cover a wide range of cognitive abilities and minimize floor and ceiling effects. However, their use with the elderly can be problematic because older people may have negative attitudes towards new technologies [12]. The test interface can appear intimidating and/or counterintuitive to older adults. The lack of familiarity with new technologies and the potential presence of technology anxiety could influence performance and willingness to undergo such testing in older adults. Moreover, since tests assisted by new technologies are still not widely used compared to traditional tests, the lack of normative data and psychometric standards should be noted [10]. Despite the proliferation of laboratory- and internet-based computerized cognitive assessment platforms and their many advantages, these systems are still not as widely used as many classic paper-and-pencil tests, particularly in the older adult population [9]. It is therefore necessary to develop a tool that combines the positive aspects of current new technology tests (e.g., sensitivity, accuracy, standardization) without creating attrition, especially with computer-based ones. Wireless Lighting Systems (WLSs), like *BlazePod*, *Fitlight*, or *Witty SEM* (Microgate), are a potential solution. They consist of multiple light systems programmed to light up in different colors. In addition to emitting different colors, the *Witty SEM* system can vary shapes (e.g., letters, numbers, icons). *Witty SEM* can be used as a start light, with or without a countdown, to manage timed starts, and as an agility test. For about ten years, it has mainly been used to assess the reactive agility of athletes, like handball players [13], table tennis players [14], or basketball players [15], or used as a training method [16]. However, *Witty SEM* can also be used to assess and train cognitive functions. Still, only some studies have used it to research the effect of aging on cognition. WLSs could be used to create tests that are (a) cognitive with motor stimulation, (b) ecological, and (c) sensitive.

- **Cognitive with motor stimulation**

What makes *Witty SEM* interesting is the ability to create a test that appeals to motor and cognitive skills. At the cognitive level, it is possible to choose various testing options with different shapes and colors and, therefore, multiple cognitive difficulties. At the physical level, the participants must perform a motor task (e.g., hitting the target as quickly as possible) to confirm cognitive processing (e.g., finding the right target to hit). These new cognitive tests with motor stimulation could be helpful in studies evaluating the effects of physical activity programs. Moreover, the original, colorful character, supported by technology, could suggest a gaming spirit, so *Witty SEM* could be useful for studies of cognitive training programs with video games or exergames.

- **Ecological value**

Motor and cognitive tasks would be combined to perform the tests, making them more ecological. Several noncognitive factors, including motor functioning, can influence the relationship between test performance and everyday performance [17]. Dual-task performance assessment is clinically important as it represents a real-life condition for performing daily activities, and can better describe the potential for adverse events such as falls [18,19]. Ecological tests, associating physical and cognitive aspects, could assess the cognitive impact on everyday tasks more realistically. In daily life, older adults often have to perform a physical task with a cognitive demand (e.g., making food, walking to the right place to avoid obstacles, reacting quickly).

- **Measurement sensitivity**

The central control is provided by a stopwatch thanks to a radio or a Bluetooth transmission system and records data in real-time. Response latencies with millisecond accuracy lead to greater potential test sensitivity, making *Witty SEM* a valuable tool.

Effective evaluation methods to reduce the risk of physical and cognitive impairment are crucial. WLSs, especially *Witty SEM*, could enable researchers to investigate changes that may not be detected using conventional methods, and to assess and follow cognitive changes in aging over the long term. This study’s originality also lies in using this system to measure the effect of age compared with traditional cognitive tests.

The purpose of this pilot study was (1) to test whether cognitive tests designed with a WLS were sensitive to the effects of age on cognition in the same way as traditional tests and (2) to test the convergent validity of WLS tests. We hypothesize that the WLS tests will show differences in performance by age and that their scores will be highly correlated with those of traditional tests. To study this, we designed three tests to assess four main cognitive functions (inhibition, flexibility, visuospatial short-term memory, visuospatial working memory), and we used three of the most useful traditional tests (*Spatial Span Test*, *Stroop Test*, *Trail Making Test*).

## 2. Materials and Methods

### 2.1. Population

Recruitment of participants was conducted between October 2022 and January 2023. Potential participants were contacted via mail, flyers, and the Risc website (a CNRS service unit). A power analysis, using G*Power version 3.1.9.7, based on the primary statistical test planned for this study (*t*-test independent group) revealed that a sample size of 32 participants was needed (α = 0.05; power = 0.80; effect size: d = 0.9). As a very high effect size was expected (i.e., d > 0.9 [20]) for differences between young and old, it was set on this value: d = 0.9. All participants met the following inclusion criteria: (a) engage in less than 2.5 h of physical activity per week, (b) score 3 or higher on a subjective health scale (1 = very poor to 5 = excellent), (c) have no major pathology, (d) have normal or corrected vision and hearing, (e) have no difficulty bending or using their shoulders, and (f) not play video games or exergames. The older adults group (OLD) participants should be over 60 years of age, and those of the young adults group (YOUNG) should be between 18 and 30 years of age. All gave their written informed consent and were not compensated for their participation.

### 2.2. Outcomes

The tests were conducted individually for one hour in a quiet room in the University Paris-Cité (France) laboratory.

#### 2.2.1. Wireless Lighting System (WLS) Cognitive Tests

**Figure 1:**
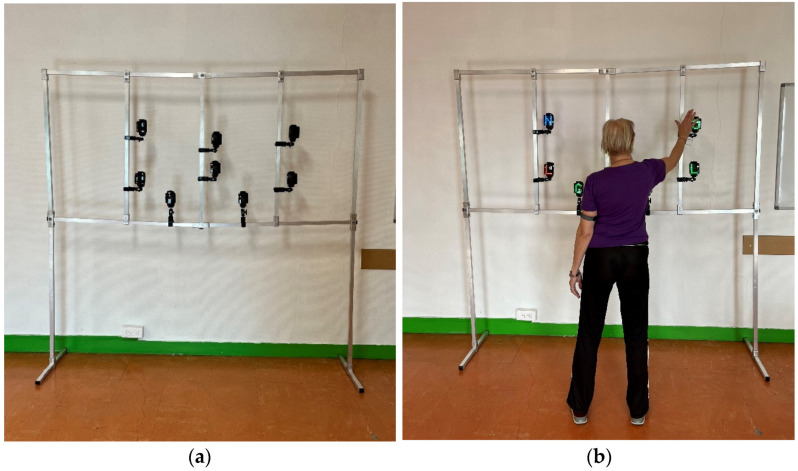
(**a**) Specially designed rack; (**b**) participant taking the WLS inhibition test.

The WLS tests were performed with *Witty SEM*, with a set of eight 5 cm × 7 cm semaphores equipped with an LED matrix—on which various colors and shapes can be displayed. Witty semaphores were placed individually (2 in the center and 6 in the periphery) on a specially designed rack (Figure 1). The tests were initiated by a wireless control panel operated by radio transmission. The Witty Manager software enables the researchers to create new tests and record and save collected results. Three tests were designed. For each test, the participant stood in the middle of the rack, and at arm’s length from the semaphores. They had to turn off the lights that appeared as fast as possible, using their dominant hand, without moving their feet, and returning their hand to the middle of their hips between each repetition. Witty semaphores include a motor sensor; it is thus necessary to approach it to turn it off. For each test, a trial with 5 examples was carried out.

These tests, which combine physical and cognitive aspects, offer a less isolated cognition assessment. The standing position and the obligation to confirm cognitive processing with physical action contribute to providing an ecological assessment closer to the constraints of daily life (e.g., performing several tasks simultaneously, time pressure).

- **The WLS Inhibition Test.**

**Figure 2:**
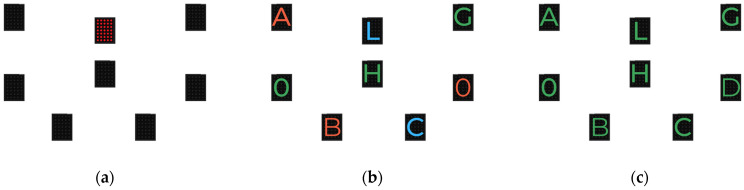
WLS inhibition tests: (**a**) part 1: Reaction Time; (**b**) Part 2: Reaction Time with Simple Inhibition; (**c**) part 3: Reaction Time with Complex Inhibition.

This test is separated into 3 parts. In the first part (Figure 2a), *Reaction Time (RT)*, the participant had to turn off the semaphores that randomly lit up red as fast as possible. The others remained unlit. In the second part (Figure 2b), *Reaction Time with Simple Inhibition (SI)*, the participant had to turn off the randomly lit semaphores with the green letter O as fast as possible. The others lit up with other colors (i.e., blue, red) associated with a letter (i.e., A, B, C, D, E, F, G, H). In the last part (Figure 2c), *Reaction Time with Complex Inhibition (CI),* the participant had to turn off the randomly lit semaphores with the green letter O as fast as possible. The others lit up in green, associated with another letter (i.e., A, B, C, D, E, F, G, H). Each part consisted of 20 trials; each trial started 1500 ms after the previous response. The total time to complete each part was recorded in milliseconds. Inhibition was highlighted by analyzing the time difference in the CI–RT result.

- **The WLS Spatial Span Test.**

**Figure 3:**
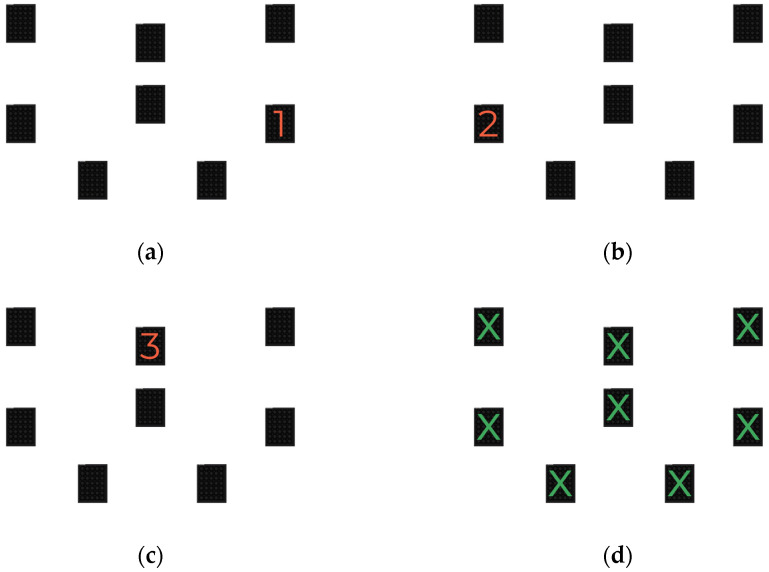
WLS Spatial Span Test: (**a**–**c**) part 1; (**d**) part 2.

In the first part, which assesses visuospatial short-term memory, an ascending numerical sequence (e.g., 1, 2, 3, 4) was displayed thanks to the semaphores that lit up one after the other at the rate of one per second (Figure 3a–c). When all semaphores showed a green dot (Figure 3d) at the end of the sequence, the participant had to indicate the sequence of the numbers in the same order. In the second part, which assesses visuospatial working memory, a descending numerical sequence (e.g., 4, 3, 2, 1) was displayed thanks to the semaphores that lit up one after the other, at the rate of one per second. When the semaphores showed a green dot at the end of the sequence, the participant had to indicate the sequence of the numbers in the reverse order. For each part, the tests started with a sequence of 2 numbers. An additional number was added to the series for every two successful attempts. One point was awarded for each successful attempt, with 10 attempts.

- **The WLS Flexibility Test.**

**Figure 4:**
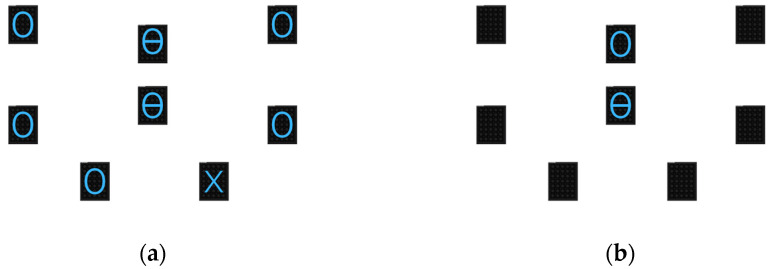
WLS flexibility test: (**a**) part 1; (**b**) part 2.

Two semaphores (A and B), either those where the “circle with a horizontal line” or “the circle with a vertical line” symbols appeared, were placed near each other in the middle of the frame. The other semaphores were placed in a circle around the central ones to create “sectors” where the peripheral stimulus symbol would be recognized. The test was divided into 2 parts:

On the central semaphores A and B, the participant was shown a symbol—which was the same for both semaphores—corresponding to two icons (“circle with a horizontal line” or “the circle with a vertical line”). The participant had to remember this symbol. In addition, a symbol appeared (a blue cross) on one of the other peripheral semaphores, while other symbols appeared (a blue circle) on the other peripheral semaphores (Figure 4a). The participant had to remember the location of the cross. All information remained displayed for 1.995 s.

Once all the lights were off, the central semaphores offered two options: a circle with a horizontal line or a circle with a vertical line. The participant had to hit the correct initial symbol and the target on the peripherical semaphores where the blue cross was.

One point was awarded for each successful attempt, with 10 attempts.

#### 2.2.2. Traditional Cognitive Tests

- **The Spatial Span Test**

This is a subtest of the Wechsler Memory Scale [7]. The first part assesses visuospatial short-term memory. The participant had to reproduce a sequence of cubes designated by the examiner in the same order. In the second part, which evaluates visuospatial working memory, the participants reproduced a sequence of movements in reverse order. The number of cubes progressively increased, determining the *spatial span* and the *backward spatial span*. For each level, the participants completed 2 trials, scoring 1 point for each successful attempt. The test ended when two errors were made at the same level.

- **The Stroop Test** [6]

The Stroop Test was used to assess inhibition. Each board contained 100 items divided into 10 columns. Only the first 3 boards of the 4 were kept: the *naming condition* contained colored rectangles (color board); the *reading condition*, with color words appearing in black ink (word board); and the *interference condition* contained color words, appearing in color different from their meaning (color-word board). In all conditions, the performance measure was the number of correct responses in 45 s. The interference score was calculated as follows:

Interference score = color-word board score/(mean of color board and word board scores).

- **The Trail Making Test (TMT)** [5]

The TMT measures cognitive flexibility. Part A consisted of linking a series of increasing numbers from 1 to 25 by selecting the appropriate digit. In part B, the participant connected 2 series of symbols in alternation: 1 of numbers and 1 of letters. This had to be completed as quickly as possible without lifting the pen from the paper. If mistakes were made, the examiner informed the participant, who had to correct them. Flexibility was highlighted by the time difference in result B-A, a purer measure of flexibility.

### 2.3. Data Analysis

The analyses were performed using JASP software. Descriptive data (i.e., mean, standard deviation, range) were reported for all variables. Before conducting the inferential statistical tests, all relevant assumptions were checked (normal distribution, homogeneity of variance of independent variables). The results are provided at The results are provided in Tables S1 and S2, https://osf.io/s4k8u/ (accessed on 13 November 2023). Parametric tests were used—if relevant assumptions were met—and nonparametric tests were employed if relevant assumptions were not met. Mann–Whitney tests analyzed differences between groups. To determine the relationships between tests, Spearman’s or Pearson’s correlation coefficients were used. All statistical analyses with a *p*-value < 0.05 were considered significant. Effects sizes, rank-biserial correlation (*r*) for Mann–Whitney U-test, and Spearman’s rho (ρ) for Spearman’s correlation or Pearson’s r were calculated to study the power of the results. As indicated in the effect size guidelines from Cohen [20], *r* and ρ are considered low if the effect size is between 0.10 and 0.30, medium if it is between 0.30 and 0.50, and large if it is superior to 0.50.

## 3. Results

### 3.1. Participants

Thirty-three subjects participated in the study: 15 young adults (9 women) and 18 older adults (12 women). Individual variables (i.e., age, BMI, height, subjective health) are given in Table 1. There was no statistical difference (*p* > 0.05) between YOUNG and OLD for height.

The scores for the WLS and traditional (TRAD) tests for YOUNG and OLD groups are presented in Table 2. There was a ceiling effect on WLS flexibility tests for the YOUNG group. The ranges for all the other tests were sufficient, with no sign of a floor or ceiling effect.

### 3.2. The Ability of the Tests to Detect the Effects of Aging

The first step of data analysis involved examining the ability of the WLS tests to discriminate between the YOUNG and OLD groups. The results for the group effect on the WLS and TRAD tests for the YOUNG and OLD groups are given in Table 2. There was a significant difference between the YOUNG and OLD groups on all six WLS test measures (*p* < 0.01 or *p* < 0.001) with large effect sizes (*r* > 0.50), and all TRAD measures with medium to large effect sizes. Statistical analysis could not be performed for the flexibility of the WLS tests because the variance was equal to 0 for the YOUNG group.

### 3.3. Convergent Validity

In the second step of data analysis, the convergent validity of the tests was assessed by calculating correlations between the WLS and the TRAD tests separately for the YOUNG and OLD groups (Table 3). For the OLD group, there was a high correlation between two measures of inhibition, *Complex Inhibition (WLS)* and *color-word board (TRAD)* (*r* = −0.73, 95% CI = −0.89; −0.41, *p* < 0.001) and a moderate correlation between the two others, *inhibition score (WLS)* and *interference score (TRAD)* (*r* = −0.49, 95% CI = −0.78; −0.03, *p* < 0.05). There were high correlations between the two measures of flexibility, the *flexibility test (WLS)* and *Time B–Time A (TRAD)* (ρ = −0.63, 95% CI = −0.85; −0.24, *p* < 0.01) and between the two measures of working visuospatial memory, the *visuospatial working memory (WLS)* and *backward spatial span (TRAD)* (*r* = 0.67, 95% CI = 0.29; 0.86, *p* < 0.01). There was no correlation between the two measures of visuospatial short-term memory, the *visuospatial short-term memory (WLS)* and *spatial span (TRAD)* (*p* > 0.05).

For the YOUNG group, there was no correlation between measures (*p* > 0.05). Statistical analysis could not be performed for the flexibility test results because the variance was equal to 0.

## 4. Discussion

While much research is being conducted to find optimal strategies for improving senior citizens’ physical and cognitive health, assessment methods remain conventional. It is necessary to develop new tests that are more ecological and precise than the old ones.

The first aim of this pilot study was to check whether cognitive tests designed with a WLS were sensitive to the effects of age in the same way as traditional tests. WLS tests enable researchers to see significant differences between young and older adults on several cognitive functions (i.e., inhibition, visuospatial short-term and working memory) with high effect sizes. As is the case in the literature, significant differences in performance were also found between YOUNG and OLD on traditional cognitive tests [21,22,23]. Statistical analysis could not be performed for the WLS flexibility test scores because of the ceiling effect of the YOUNG group. This may be caused by the fact that performance is recorded with a score rather than a time. Indeed, in this experiment, to compare the WLS test scores to their traditional equivalents, the WLS tests were programmed to be nonadaptive to the individual’s abilities. If this tool were used to assess cognitive functions in young adults, the tests would have to be adaptive or initially more complicated.

The second aim of this study was to investigate the convergent validity of the WLS tests, assessed by examining whether the tests measured the desired cognitive functions. For the OLD group, the correlations were moderate to high for four cognitive functions, except for visuospatial short-term memory. Confidence intervals of 95% were large (up to 0.75 for correlations between interference score and inhibition score). Therefore, it would be necessary to have a larger sample to be able to reduce the 95% CI. WLS makes substantially assessing the desired functions possible and may reveal the daily living abilities of older adults more than traditional tests. Nevertheless, as Zygouris and Tsolaki’s review [10] indicated, it should be noted that equivalence between paper-and-pencil tests and their computerized adaptations cannot be taken for granted, as differences in instructions, stimulus presentation, and response methods can affect test results in older adults and even the cognitive domains and processes they actually measure. Here, the difference lies mainly in that motor realization to perform the cognitive task was essential to pass these tests. This may explain why some of the variance in the new tests was not defined by the variance in the traditional tests. Moreover, traditional tests, which can be criticized for their lack of sensitivity and accuracy, were still used to investigate convergent validity. As the WLS tests would not present these negative aspects, this also justifies why not all the variance was explained. Currently, traditional tests are the gold standard, and the most widely recognized tests for cognitive assessment, with standards for older adults.

For the YOUNG group, no correlation between measures was found. The tests were perhaps too easy for the young and did not successfully assess the desired cognitive functions. With young people, there should be more semaphores, stimuli should appear more quickly, and motor and cognitive difficulties should be amplified. Knowing that the performances of motor and cognitive functions are highly linked in the elderly [24], it is necessary to develop tests that are closer to the challenges of everyday life, rather than only an isolated cognitive task. Since traditional tests lack ecological validity, this makes WLS tests a promising alternative. Ecological validity is “the degree to which test performance corresponds to real-world performance” [17]. Therefore, these new tests should not be considered as equivalents to traditional tests but as ecological tests, combining physical stimulation with cognitive assessment (e.g., hitting the right target as quickly as possible). Even if the Witty SEM configurations do not directly mimic situations in actual daily life, the additional motor aspect makes the assessment of cognition less isolated and, therefore, closer to the multidimensional demands of daily life. The ecological value, in particular the verisimilitude (i.e., the similarity of the test to everyday tasks [25]) of the test, could be enhanced by adding more complex physical tasks, such as walking or balance situations. Increasing verisimilitude would allow tests to better capture the essence of everyday cognitive skills [17]. Moreover, new tests could be developed with this tool, including dual-task and coordination tests, which are lacking.

The main limitation of this study is the inability to perform inferential statistics to test the differences between the two groups for the WLS flexibility test. It would be interesting to modify the test and make it scalable (e.g., increase the speed and difficulty) according to the participant’s level. Moreover, the results were compared only for three cognitive tests. Other tests could be created to evaluate planning, attention, or long-term memory. It would be interesting to test a larger panel of the elderly population to obtain normative data and psychometric standards. The convergent validity of these tests has been proven for older adults. Now, reliability aspects need to be studied, particularly test–retest reliability and inter-rater reliability.

This first pilot study showed encouraging results, which must be confirmed by a more comprehensive study with a larger sample. WLS tests could be compared with other cognitive tests, particularly ecological tests, such as some BADS tests [26]. WLS tests appear to be valid in assessing cognitive functions (i.e., flexibility, visuospatial short-term and working memory, inhibition) in a physical situation for older adults, and are also sensitive to age effects. WLS tests can evaluate older adults’ cognition, making the assessment of cognition less isolated and, therefore, more ecological. This could also align with the multimodal intervention programs emerging in the literature and offer more complete transfer effects with real life.

## Figures and Tables

**Table 1:** Means of age, BMI, height, and subjective health for YOUNG and OLD groups.

	YOUNG		OLD	
	M	SD	M	SD
Age (*n*)	23.47	2.13	71.44	5.40
BMI (m^2^/kg)	21.14	3.13	24.71	2.92
Height (cm)	169.00	10.04	165.67	10.47
Subjective health ^1^ (*n*)	4.27	0.46	3.83	0.51

Abbreviations: BMI = Body Mass Index; cm = centimeter, M = mean; *n* = number; SD = standard deviation. ^1^ Scoring: 1 = very poor, 2 = poor, 3 = good, 4 = good, 5 = excellent.

**Table 2:** Differences in TRAD and WLS tests between YOUNG and OLD.

	YOUNG		OLD				
	M (SD)	Range	M (SD)	Range	*W*	*p*	*r*
**WLS tests**							
**WLS Inhibition**							
*Reaction time (s)*	38.54 (1.02)	37.08–40.53	41.06 (2.31)	36.82–47.63	234.00	<0.001	0.73
*Simple inhibition (s)*	49.89 (3.27)	45.03–58.24	57.08 (6.71)	46.32–67.54	217.00	<0.01	0.61
*Complex inhibition (s)*	56.50 (5.18)	50.51–66.18	64.75 (6.76)	52.93–74.75	224.00	<0.001	0.66
*Inhibition score (s)*	17.96 (4.77)	13.13–26.37	23.69 (6.00)	13.67–32.47	213.00	<0.01	0.58
**WLS Spatial Span Test**							
*Visuospatial short-term memory (n)*	8.60 (1.12)	7–10	7.28 (0.89)	6–9	54.00	<0.01	0.60
*Visuospatial working memory (n)*	8.20 (0.94)	6–9	6.61 (0.85)	5–8	32.50	<0.001	0.76
**WLS Flexibility**							
*Flexibility (n)*	10.00 (0.00)	10–10	7.11 (3.16)	0–10	-	-	-
**TRAD tests**							
**Spatial Span Test**							
*Spatial span (n)*	8.47 (1.96)	5–12	6.39 (1.42)	4–9	54.50	<0.01	0.60
*Backward spatial span (n)*	8.47 (2.10)	5–12	5.56 (1.85)	3–8	43.00	<0.001	0.68
**Stroop**							
*Color board (n)*	122.00 (17.30)	95–152	106.00 (22.76)	63–160	61.50	<0.05	0.52
*Word board (n)*	83.57 (13.96)	60–113	71.89 (25.18)	33–150	70.50	<0.05	0.44
*Color-word board (n)*	63.86 (13.94)	40–98	45.94 (27.03)	18–139	38.50	<0.001	0.69
*Interference score (n)*	0.62 (0.14)	0.47–1.03	0.50 (0.17)	0.24–1.02	49.00	<0.01	0.61
**TMT**							
*Time A (s)*	14.15 (4.09)	10.33–24.33	31.78 (13.98)	15.01–62.06	255.00	<0.001	0.89
*Time B (s)*	33.32 (9.57)	21.05–24.75	93.04 (62.95)	32.73–227.99	245.00	<0.001	0.81
*Time B–Time A (s)*	19.16 (9.09)	0.73–35.57	61.27 (51.94)	14.91–167.90	212.00	<0.01	0.57

Abbreviations: M = mean; n = number; SD = standard deviation; s = second; TMT = Trail Making Test; TRAD = traditional; WLS = Wireless Lighting System.

**Table 3:** Convergent validity between TRAD and WLS tests.

	WLS		Complex Inhibition		Inhibition Score		Flexibility		Visuospatial Short-Term Memory		Visuospatial Working Memory	
TRAD												
			YOUNG	OLD	YOUNG	OLD	YOUNG	OLD	YOUNG	OLD	YOUNG	OLD
**Color-word** **board**		*r/*ρ	−0.41	**−0.** **73**								
		95% CI	−0.77–0.16	**−0.89–−0.41**								
		*p*	0.14	**<0.001**								
**Interference** **score**		*r/*ρ			−0.27	**−0.49**						
		95% CI			−0.70–−0.30	**−0.78–−0.03**						
		*p*			0.34	**0.04**						
**Time B-A**		*r/*ρ					X	**−0.63**				
		95% CI					X	**−0.85–−0.24**				
		*p*					X	**0.005**				
**Spatial span**		*r/*ρ							0.45	0.05		
		95% CI							−0.08–0.78	−0.43–0.50		
		*p*							0.09	0.84		
**Backward spatial span**		*r/*ρ									0.46	**0.67**
		95% CI									−0.07–0.78	**0.29–0.86**
		*p*									0.09	**0.002**

Abbreviations: TRAD = traditional; WLS = Wireless Lighting System; X = statistical analysis could not be performed; 95% CI = 95% confidence interval. Bold values are significant.

## Data Availability

The data presented in this study are available in the Open Science Framework at https://osf.io/s4k8u/ (accessed on 15 November 2023).
